# The Associations Between Workplace Bullying and Physical or Psychological Negative Symptoms: Anxiety and Depression as Mediators

**DOI:** 10.5964/ejop.v15i4.1733

**Published:** 2019-12-19

**Authors:** Alessandro Lo Presti, Paolo Pappone, Alfonso Landolfi

**Affiliations:** a Dipartimento di Psicologia, Università degli Studi della Campania “Luigi Vanvitelli”, Viale Ellittico, Caserta, Italy; b ASL Napoli 1 Centro, Ambulatorio Sovradistrettuale Mobbing e Disadattamento lavorativo, Napoli, Italy; Webster University Geneva, Geneva, Switzerland

**Keywords:** workplace bullying, depression, anxiety, health, well-being, physical symptoms, psychological symptoms

## Abstract

Workplace bullying is a critical issue for its negative consequences on victims’ health and well-being. This study aimed at examining the intermediate roles of anxiety and depression, in the relations between workplace bullying as a predictor, and physical and psychological negative symptoms as outcomes. In particular, it was hypothesized that workplace bullying would be associated with higher anxiety and depression and, through them, with higher physical and psychological negative symptoms. We sampled 151 Italian employees, who called on a workplace bullying public clinical center as victims and filled a paper-and-pencil questionnaire. Results of bootstrapped regressions showed that only anxiety mediated the association of workplace bullying with negative physical symptoms while both anxiety and depression mediated its association with negative psychological symptoms. The results have implications for the development of appropriate intervention strategies for both prevention and clinical treatment. In particular, timely diagnosing and treating anxiety and depression could prevent subsequent problems related to psychological and physical symptoms such as colitis, headache, tiredness, nervousness, etc. Organizational interventions in terms of primary prevention are also discussed. From an empirical standpoint, the study contributed to disentangling the differential roles of anxiety and depression with respect to physical and psychological symptoms; moreover, overcoming a common limit of workplace bullying research, the current study was carried out on actual victims.

Workplace bullying is a compelling psychosocial issue for its individual, organizational, and social negative effects, representing a complex multicausal psychosocial phenomenon that involves a series of factors found at many explanatory levels (Burke, Mathiesen, Einarsen, & Fiksenbaum, 2008; Einarsen, 1999; Einarsen, Hoel, Zapf, & Cooper, 2011a; Hoel & Cooper, 2001; Zapf, 1999). Workplace bullyling stands for the severe form of harassing people in organizations and, theoretically, it is an extreme form of social stressor at work. Apart from individual and organizational effects, workplace bullying involves significant financial costs that can impair organizational functioning and impact on social welfare sustainability; in fact, it has been calculated that for every single victim there are historical costs for sickness absence of 125,000 British pounds (Kivimaki, Elovainio, & Vahteera, 2000) while it was extimated a total expenditure of 3.7 billion pounds only in the United Kingdom (Beswick, Gore, & Palerman, 2006). Generally speaking, the prevalence of workplace bullying shows a large variation depending on how it is measured, in fact Nielsen, Matthiesen, and Einarsen (2010) found a general estimated prevalence rate for workplace bullying ranging from 11 to 18%, this variation due to the measurement method used. Moreover, with the use of meta-analytical evidence, the same authors estimated that at least 15% of the global working population was exposed to negative workplace behaviors that can be experienced as bullying.

Exposure to workplace bullying and its associations with health and well-being have been largely examined; among the others: psychological disorders, depression, burnout, anxiety and aggression, as well as psychosomatic and musculoskeletal disorders (Björkqvist, Österman, & Hjeltbäck, 1994; Einarsen & Raknes, 1997; Niedl, 1996; Zapf, Knorz, & Kulla, 1996). However, studies examining the reciprocal relations between clusters of variables consequent to workplace bullying have been largely overlooked. Building on this, this paper aims at examining the associations between workplace bullying as a predictor, anxiety and depression as intermediate variables, and psychological and physical negative symptoms as final outcomes. Disentangling the potential mediated effects from direct effects could be useful in targeting anxiety and/or depression through appropriate treatment among workplace bullying victims in case mediated effects were proved or else focusing on alternative treatments directly focused on physical and/or psychological negative symptoms if mediation was not proved. Additionally, while most studies had been usually carried out within organizational contexts with heterogeneous samples, data for this study have been collected in an Italian clinical center dedicated to workplace bullying victims, attesting over 20 years of clinical practice in terms of prevention and treatment, thus providing evidence on actual victims of workplace bullying as well as on an under-represented country in workplace bullying research as Italy.

## Theoretical Foundation

“Bullying at work means harassing, offending, or socially excluding someone or negatively affecting someone’s work” (Einarsen, Hoel, Zapf, & Cooper, 2011b, p.39). A single conflict can not be called workplace bullying if the incident is an isolated event or if two parties of approximately equal strength are in conflict (Einarsen, Hoel, Zapf, & Cooper, 2003). Leymann (1990, 1996) suggested, as a time criterion to characterize bullying as a severe form of social stress, that such events should occur at least once a week for more than 6 months. Others have used repeated exposure to negative behaviours within a 6-month period as the proposed timeframe (Einarsen & Skogstad, 1996). Hence it is clear that while many examples of interpersonal aggression take the form of single episodes, bullying in the workplace is characterized by a systematic and prolonged exposure to repeated negative and aggressive behavior also and more of a psychological nature, including non-behavior and acts of social exclusion (Einarsen et al., 2011b; Leymann, 1996).

Several theoretical models explaining the relations between workplace bullying and different clusters of antecedents and consequences are available. Among others, Nielsen and Einarsen (2012) advanced a theoretical model integrating insights from Transactional stress theory (Lazarus & Folkman, 1984), Affective Events Theory (AET; Weiss & Cropanzano, 1996) and Cognitive Activation Theory of Stress (CATS; Ursin & Eriksen, 2004). According to their model, exposure to systematic and prolonged aggressive behaviors negatively affects the targeted individual and the effects of exposure to workplace bullying can initially be explained by cognitive mechanisms: in particular, individuals recur to evaluation processes which allow to interpret external and/or internal triggers in terms of more or less threat to the individual, based on the current evaluation of available resources; these appraisal processes can lead to cognitive discrepancy when triggers are perceived as unmanageable, thus leading to the feeling of being unable to face the current situation (i.e., self helplessness). Therefore, the negative effects are triggered by repeated and chronic cognitive activation of a prolonged bullying process. Cognitive activation would also lead to a prolonged physiological activation which will subsequently manifest itself through the impairment of health and well-being (Ursin & Eriksen, 2004), decreased sleep quality, increased levels of cortisol, increased heart rate and increased mortality (Meurs & Perrewe, 2011; Ursin & Eriksen, 2004). More in detail, Nielsen and Einarsen (2012) differentiated between job-related outcome variables (attitudes and behaviour) and outcome variables related to health and well-being. With regard to this latter cluster, they identified several distinct variables: mental health problems (thus differentiating between anxiety and depression), physical health problems, post-traumatic stress disorder, burnout, sleep problems, strain, etc. Mikkelsen and Einarsen (2002) argued that “exposure to bullying behaviors at work may alter the victims’ emotional state and their views of themselves and others, this in turn causing higher levels of reported psychological health complaints and psychosomatic complaints” (p. 398).

From the point of view of psychological symptomatology, Brodsky (1976) identified, in addition to the physical symptoms, both depressive reactions and symptoms related to depression (e.g., impotence, lack of self-esteem, insomnia), and reactions related to psychological symptoms (e.g., hostility, hypersensitivity, loss of memory, feelings of victimization, nervousness, avoidance of social contact). Based on this, in the present paper anxiety and depression were differentiated with respect to physical negative symptoms (e.g., high blood pressure, colitis, back pain, headache) and psychological negative symptoms (e.g., sleep problems, disinterestedness, loss of concentration, tension). As anticipated before, our aim was to differentiate between these outcomes, furthermore examining if anxiety and depression may act as intermediate variables thus mediating the effects of workplace bullying on psychological and physical negative symptoms. Available evidence and theoretical underpinning supporting our study hypotheses will be detailed in the next section.

## Study Hypotheses

Available evidence suggests that bullying at work is a powerful stressor that affects, among other things, the health and well-being of individuals. According to Nielsen and Einarsen (2012), exposure to systematic and prolonged aggressive behaviors negatively impacts on the individual causing a cognitive activation and a subsequent physiological activation that, if prolonged, will lead to the impairment of health and well-being. In particular, given that each subject can have different consequences and react differently, leading to alterations in cognitive-emotional balance (e.g., depression, anxiety, obsessions, panic attacks, emotional anesthesia) as well as in the psychosomatic balance (e.g., gastrointestinal disorders, headache, dizziness, arterial hypertension, disorders of sexuality and sleep, etc.), it appears compelling to examine the differential effects on the above mentioned outcomes.

As for the cognitive-emotional effects, being a victim of systematic and intentional psychological harm by other individuals seems to produce severe reactions such as depression and anxiety, negative effects triggered, according to Nielsen and Einarsen's Model (2012), by repeated and chronic cognitive activation of a prolonged bullying process. In fact, among the different consequences of workplace bullying, higher levels of psychological disorders, not only anxiety and depression, but also burnout and aggression, have been found (Björkqvist et al., 1994; Einarsen & Raknes, 1997; Niedl, 1996; Zapf et al., 1996). Brousse et al. (2008) have found higher levels of stress and anxiety–depression disorder developed by targets of workplace bullying at 12-month post-consultation. Bilgel, Aytaç and Bayram (2006) found that workers who had reported bullying showed lower levels of job satisfaction and higher levels of work-related stress, as well as higher scores of anxiety and depression than those who had not reported bullying. According to these authors, the significant association between bullying and subsequent depression suggests that bullying is an etiological factor for subsequent mental health problems. Therefore, it appears that being a victim of repeated adverse behaviors can lead to increased anxiety and depressive symptoms, given that the working environment, and in particular social relationships within it, may be perceived as threatening and unpredictable. Based on available evidence, we expect that:

H1: Exposure to workplace bullying is positively associated with an increased presence of anxiety (a) and depression (b);

As mentioned above, workplace bullying at work has been linked to impaired health and well-being. With regard to the latter, that is the abovementioned psychosomatic balance, previous research has focused not only on the increase in anxiety or depressive symptoms but has also on psychological and physical negative symptoms. According to Djurkovic, McCormack, and Casimir (2003), workplace bullying was positively associated with physical symptoms, negative affect, and with intention to leave the job. Their results showed that bullying led to negative affect which in turn led to physical health problems. As already mentioned, according to the Nielsen and Einarsen's Model (2012), exposure to systematic and prolonged aggressive behavior causes a physiological activation that if prolonged triggers a series of physical and psychological negative symptoms. More than 40 years ago, Brodsky (1976) identified three patterns of effects on the victims. Some subjects developed vague physical symptoms, such as pains, loss of strength, chronic fatigue, weakness and various aches. Some other subjects reacted with depression and depression-related symptoms (e.g., impotence, lack of self-esteem, and sleeplessness), while others subjects, instead, reacted with psychological symptoms, such as hostility, hypersensitivity, loss of memory, feelings of victimization, nervousness, and avoidance of social contact. As mentioned above, experiencing an unmanageable cognitive dissonance between what is expected and what actually happens is associated with persistent levels of stress and disease, such as decreased sleep quality, increased levels of cortisol, increased heart rate and increased mortality (Meurs & Perrewe, 2011; Ursin & Eriksen, 2004). Thus, it is likely that individuals, apart from more specific anxiety and depressive symptoms, will develop other more general negative symptoms related both to the physical and psychological domain. Therefore, it is expected that:

H2: Exposure to workplace bullying is positively associated with an increased presence of physical (a) and psychological (b) negative symptoms;

This paper aimed not only at testing direct associations between workplace bullying and the abovementioned outcome variables (i.e., depression, anxiety, physical and psychological negative symptoms), but it is proposed that anxiety and depression may act as intermediate variables between workplace bullying and physical and psychological negative symptoms. Besides anxiety and depression, other common symptoms such as musculoskeletal disorders have been reported by victims in several European countries as a result of bullying at work (Einarsen & Raknes, 1997; Niedl, 1996; O'Moore, Seigne, McGuire, & Smith, 1998; Zapf et al., 1996). Brousse et al. (2008) have found that many patients, at 12-month post-consultation, reported various persistent somatic symptoms or disorders including: appetite and digestive disorders, weight gain, palpitations, migraines, giddiness and poorly systematized arthralgia or muscular pain. Trivedi (2004) found that a high proportion of patients with depression seeking treatment in a primary care setting had only physical symptoms, in fact physical pain and depression would seem to have a biological connection deeper than the simple cause and effect (see also O’Neil, 2013). Kivimäki et al. (2003) found a higher risk of cardiovascular disease due to prolonged bullying targets and a higher risk of developing depressive symptoms. Moreover, Henningsen, Zimmermann, and Sattel (2003) found, through a meta-analytical method, a moderate and significant association between unexplained physical symptoms, anxiety and depression. Other scholars have also pointed out that experience of depression (Beekman et al., 2002) and anxiety (De Beurs, Beekman, Van Balkom, & Deeg, 1999) can lead to subsequent somatic and psychological complaints. From the abovementioned evidence, it appears that depression and anxiety on one side, and physical and psychological negative symptoms on the other are related, with the former preceding and activating the latter (Nielsen & Einarsen, 2012). Thus, it is expected that:

H3: Anxiety will mediate the association between workplace bullying and negative physical (a) and psychological (b) negative symptoms;

H4: Depression will mediate the association between workplace bullying and negative physical (a) and psychological (b) negative symptoms;

## Method

### Procedure and Study Context

Data have been collected at the the A.S.M.D.L. (clinic center for victims of bullying and maladjustment in the workplace of ASL Napoli 1 Centro (Naples), founded in 2000. To date, this public clinical center has treated about 1,700 cases. In the last 10 years, 905 cases have been treated: 54% men, age range 20–70 years, mean age 54 years; 46% women, age range 19–71, mean age 50 years. This center offers several specialized services, both at the individual and organizational level, in order to prevent individual work difficulties and promote organizational wellbeing. Specifically, this center has several institutional tasks, including: taking charge of workplace bullying victims; cooperating with other institutions in regards to psychosocial risks prevention and intervention; workplace bullying research and prevention; assessment of psychiatric disorders and conditions of psychological distress related to work activity; suggestion of individualized therapeutic pathways and rehabilitation programs for work reintegration of workplace bullying victims; clinical-diagnostic evaluation valid for legal purposes; identification of possible protective measures by employers in the case of reported cases of workplace problems.

### Participants

67 (44.4%) men and 84 women (55.6%) participated to our study. 27 (17.9%) were blue-collars, 91 (60.3%) had lower management roles, 22 (14.6%) middle management roles, and 11 (7.3%) top management roles. Mean age was 50.1 years (*SD* 8.44). Average tenure was 14.68 years (*SD* 10.93). Average years of education was 15.07 years (*SD* 3.4). Participants filled a paper-and-pencil copy of the questionnaire at a public clinical center when they, being a victim of mobbing, requested their first consultation. Participants were included in the study sample only afterwards when, after subsequent consultations, their workplace bullying status had been ascertained.

### Measures

#### Predictors

##### Workplace bullying

Workplace bullying (Citro & Pappone, 2009; Ufficio Attività Istituzionali della Sovrintendenza Medica Regionale della Campania, 2012) refers to the frequency the respondent suffered a series of negative behaviors at the workplace. It was assessed through 21 items (e.g., “feeling isolated by his/her co-workers”) with a 5-point frequency scale (0 = never, 4 = very often). Cronbach’s alpha was .83.

##### Control variables

Age, gender (1 = male, 2 = female), and occupational status (1 = blue collar, 2 = lower management role, 3 = middle management role, 4 = top management role) were inserted as control variables. This is consistent with previous evidence showing that older employees (Einarsen & Skogstad, 1996), women (Campanini, Punzi, Carissimi & Gilioli, 2006), and employees in non-managerial positions (Lange, Burr, Conway & Rose, 2019) are more frequently subject to workplace bullying.

#### Mediators

##### Anxiety

Anxiety (Conti, 1999; Zung, 1971) refers to how often the respondent felt anxious during the past several days. It was assessed through 20 items (e.g., “I feel afraid for no reason at all”) with a 4-point frequency scale (1 = never, 4 = very often). Cronbach’s alpha was .86.

##### Depression

Depression (Innamorati et al., 2006; Zung, 1971) refers to how often the respondent felt depressed during the past several days. It was assessed through 20 items (e.g., “I feel hopeful about the future”) with a 4-point frequency scale (1 = never, 4 = very often). Cronbach’s alpha was .87.

#### Outcomes

##### Physical negative symptoms

 Physical negative symptoms (Citro & Pappone, 2009; Ufficio Attività Istituzionali della Sovrintendenza Medica Regionale della Campania, 2012) refer to how often the respondent suffered in the last 6 months of a series of somatic complaints. It was assessed through 16 items (e.g., “high blood pressure,” “headache,” “colitis,” “back pain”) with a 5-point frequency scale (0 = never, 4 = very often). Cronbach’s alpha was .83.

##### Psychological negative symptoms

Psychological negative symptoms (Citro & Pappone, 2009; Ufficio Attività Istituzionali della Sovrintendenza Medica Regionale della Campania, 2012) refer to how often the respondent suffered in the last 6 months of a series of psychological complaints. It was assessed through 14 items (e.g., “unpleasant dreams,” “concentration problems,” “tension,” “disinterestedness”) with a 5-point frequency scale (0 = never, 4 = very often). Cronbach’s alpha was .87.

### Data Analysis

Missing values (2.98% of all expected cells) for continuous variables were replaced through the Expectation Maximization method (SPSS 21).

Preliminary analyses, as mean, standard deviation and Pearson correlations were performed in order to explore the relationships between predictor, mediators and outcomes.

In order to evaluate the significance of direct and indirect effects, bootstrapping procedure with 5,000 samples with replacement from the full sample to construct bias-corrected 95% confidence intervals (hereafter 95% CI; LL = lower level of confidence interval, UL = upper level of confidence interval) was used (Preacher & Hayes, 2008). Any effect is statistically significant when zero is not included in the CI. Continuous variables were standardized before calculating regression models.

## Results

Asymmetry values ranged between −.19 (workplace bullying) and −.86 (depression). Kurtosis values ranged between .66 (depression) and −.94 (workplace bullying).

Means, standard deviations and correlations between study variables are depicted in Table 1.

**Table 1:** Descriptive Statistics and Zero-Order Correlations

	*M*	*SD*	1	2	3	4	5	6	7
1) Sex^a^			-						
2) Age			−.10	-					
3) Occupational level^b^			.06	.21**	-				
4) Workplace bullying	2.19	0.73	−.06	−.03	−.09	-			
5) Anxiety	2.9	0.48	.25**	.05	−.05	.31***	-		
6) Depression	3.01	0.5	.24**	−.01	−.01	.40***	.73***	-	
7) Physical negative symptoms	2.21	0.72	.27**	.10	−.16	.37***	.63***	.56***	-
8) Psychological negative symptoms	2.51	0.75	.11	.05	.02	.51***	.68***	.73***	63***

^a^1 = male, 2 = female; ^b^1 = blue collar, 2 = lower management role, 3 = middle management role, 4 = top management role.

**p < .01. ***p < .001.

Workplace bullying positively correlated with anxiety (*r* = .31, *p* < .001), depression (*r* = .40, *p* < .001), physical negative symptoms (*r* = .37, *p* < .001), and psychological negative symptoms (*r* = .51, *p* < .001).

Anxiety positively correlated with depression (*r* = .73, *p* < .001), physical negative symptoms (*r* = .63, *p* < .001), and psychological negative symptoms (*r* = .68, *p* < .001).

Depression positively correlated with physical negative symptoms (*r* = .56, *p* < .001), and psychological negative symptoms (*r* = .73, *p* < .001).

Finally, negative physical symptoms positively correlated with negative psychological symptoms (*r* = .63, *p* < .001).

Two regressions with boostrapping replacement were carried. In the first one, physical negative symptoms was regressed on control variables, predictor and mediators. In the second one, the outcome variable was psychological negative symptoms (Table 2).

**Table 2:** Regression Models of Negative Physical Symptoms and Negative Psychological Symptoms

	Physical Negative Symptoms		Psychological Negative Symptoms	
	B	95% CI [LL, UL]	B	95% CI [LL, UL]
Control variables		
Sex^a^	.24**	[0.06, 0.42]	−.08	[−0.24, 0.08]
Age	.01*	[0.01, 0.02]	.00	[−0.01, 0.01]
Occupational level^b^	−.14*	[−0.26, −0.03]	.05	[−0.04, 0.15]
Predictor		
Workplace bullying	.14**	[0.04, 0.23]	.19**	[0.10, 0.27]
Mediators		
Anxiety	.30***	[0.18, 0.43]	.24***	[0.12, 0.35]
Depression	.10	[−0.03, 0.23]	.31***	[0.19, 0.42]
Indirect effects		
Workplace bullying -> Anxiety	.10*	[0.04, 0.17]	.08*	[0.03, 0.14]
Workplace bullying -> Depression	.04	[−0.01, 0.09]	.13**	[0.07, 0.20]
*R*^2^	0.49***		0.63***	

*Note*. LL = lower limit; UL = upper limit.

^a^ 1 = male, 2 = female; ^b^ 1 = blue collar, 2 = lower management role, 3 = middle management role, 4 = top management role.

*p < .05. **p < .01. ***p < .001.

As for negative physical symptoms, it was positively predicted by sex (B = .24, 95% CI [0.06, 0.42]), workplace bullying (B = .14, 95% CI [0.04, 0.23]), and anxiety (B = .30, 95% CI [0.18, 0.43]), and negatively predicted by occupational level (B = −.14, 95% CI [−0.26, −0.03]). The effect of workplace bullying was only mediated by anxiety (B = .10, 95% CI [0.04, 0.17]). Explained variance by predictor and mediators was equal to 49%.

As for negative psychological symptoms, it was positively predicted by workplace bullying (B = .19, 95% CI [0.10, 0.27]), anxiety (B = .24, 95% CI [0.12, 0.35]), and depression (B = .31, 95% CI [0.19, 0.42]). The effect of workplace bullying was both mediated by anxiety (B = .08, 95% CI [0.03, 0.14]), and depression (B = .13, 95% CI [0.07, 0.20]). Explained variance by predictor and mediators was equal to 63%.

## Discussion

The present paper, consistently with Nielsen and Einarsen's Model (2012), examined the intermediate role of anxiety and depression, in the relations between workplace bullying as a predictor, and physical and psychological negative symptoms as outcomes. In particular, we examined whether workplace bullying was associated with higher anxiety and depression and, through them, with higher physical and psychological negative symptoms. Results showed the severity of the effects of bullying on mental health, which lead to more or less serious conditions of psychological/psychiatric impairment even in subjects without a significant psychiatric history.

First, we hypothesized (H1) that exposure to workplace bullying was positively associated with an increased presence of anxiety and depression. Results fully supported this hypothesis: workplace bullying was positively associated with anxiety and depression. Similar findings have been reported by Nielsen and Einarsen (2012), Brousse at al. (2008), Bilgel et al. (2006). So, exposure to systematic and prolonged aggressive behaviors could be an etiological factor for subsequent mental health problems, as anxiety and depression.

Second, given that many scholars have stressed the importance of bullying in the workplace as a predictor of physical and psychological negative symptoms, we hypothesized (H2) that exposure to workplace bullying was positively associated with an increased presence of physical and psychological negative symptoms. This hypothesis was fully supported showing that physical and psychological negative symptoms were positively predicted by workplace bullying, confirming the critical role of bullying in the onset of somatic/physical and psychological symptomatology. There results are in line with available evidence, therefore, exposure to systematic and prolonged aggressive behavior may lead to a physiological activation that triggers a series of physical and psychological negative symptoms, as confirmed by Nielsen and Einarsen's Model (2012). It derives that beyond the anxious and/or depressive symptomatology, other negative effects of workplace bullying may be found: vague physical symptoms (such as pain, loss of strength, chronic fatigue, weakness and various pains), impotence, lack of self-esteem, insomnia, hypersensitivity, loss of memory, feelings of victimization, nervousness and avoidance of social contact, decreased quality of sleep, increased heart rate.

Finally, we hypothesized (H3) that anxiety may mediate the association between bullying at work and physical (a) and psychological (b) negative symptoms and (H4) that depression will mediate the association between workplace bullying and physical symptoms (a) and negative psychological negative (b) symptoms. Results showed that only anxiety mediated the effects of workplace bullying on physical negative symptoms while, both anxiety and depression were mediators with respect to psychological negative symptoms. So, hypothesis H3 was fully confirmed: anxiety mediated the effect of bullying on both physical (H3a) and psychological (H3b) negative symptoms. Hypothesis H4 was partially confirmed given that depression mediated only the effect of bullying on psychological negative symptoms (H4b), while it did not mediate the effect of bullying on physical negative symptoms (H4a). It derives that apart from being directly associated with psychological and physical negative symptoms—at least partially—anxiety and, partly, depression represent intermediate variables through which workplace bullying potentially lead to impaired physical and psychological well-being.

## Conclusions

Workplace bullying is a psychosocial risk that can significantly impair individual well-being (Eurofound, 2017), but can also negatively impact on the organization through increased absenteeism (O’ Connell, Calvert, & Watson, 2007) and turnover (Hoel & Cooper, 2000), as well as decreased productivity (Hoel, Sparks, & Cooper, 2001). Additionally, it has been proven that it has significant financial costs for the welfare system (Beswick et al., 2006; Kivimaki et al., 2000). It derives that acquiring a better knowledge of this phenomen, its antecedents as well its consequences (at different levels), may be useful in order to prevent it and/or effectively target victimization processes and their effects.

This paper contributed to the literature providing additional evidence about the negative effects of workplace bullying on depression and anxiety (Bilgel et al., 2006; Brousse et al., 2008), as well as on physical and psychological negative symptoms (Djurkovic et al., 2003). Moreover, it was proved that the effects of workplace bullying on physical and psychological negative symptoms were mediated by anxiety and, partly, by depression. The high percentage of variance of both outcomes explained by predictors supports the salience of these factors in explaining variability in physical and psychological negative symptomatology. From a practical standpoint, it derives that timely diagnosing and treating anxiety and depression could prevent subsequent problems related for instance to increased blood pressure, pain, colitis, headache, tiredness, decreased quality of sleep, hypersensitivity, loss of memory, etc. Additionally, overcoming a common limit of workplace bullying research—that is participants not being victims of actual bullying—the participants of this study were all recruited within a public clinical center for bullying treatment and prevention, being actual victims of workplace bullying.

Our results may be useful in disentangling the intertwinement between physical and somatic symptoms, and depression and anxiety. First of all, these results may contribute to overcoming the prejudice related to the exclusivity of the "non organic" treatment; in clinical terms, it means stressing the importance of avoiding concentration, both in terms of prevention and treatment, exclusively on the organic/medical dimension but to highlight both the somatic and psychological dimension in the assessment and treatment of such disorders. Therefore, differentiating the effects on anxiety and depression from the effects on negative physical and psychological symptoms respectively, could be useful for addressing victims’ anxiety and/or depression, as well as more specific physical and/or psychological symptomatology, through appropriate and specific treatments. From a clinical point of view, having detailed information regarding the symptomatology could allow differentiating or not a possible pharmacological intervention in the treatment phase.

Although it was not the focus of the present study, significant organizational implications can be drawn from our results. In particular, consistently with available literature (Salin, 2003), the association between workplace bullying and anxiety/depression and psychological/physical negative symptoms should represent a further warning to employers to promote adequate organizational conditions that could reduce the likelihood that adverse behaviors and conflict could give rise to workplace bullying. Primary preventive interventions should target organizational culture and climate, work organization and job design, workgroups’ functioning and leadership effectiveness, reward systems and competition, just to mention a few among the main ones (Salin & Hoel, 2010).

This study suffers from a series of limitations that need to be acknowledged. A critical aspect concerns the use of a cross-sectional research design that does not allow for causal inferences; future longitudinal studies, for instance diary studies, may better examine the evolution of the phenomenon over time and, for instance, disentangle the direction of causality between anxiety/depression and physical/psychological negative symptoms. Furthermore, we only used self-report measures, therefore the relationship between the variables could be influenced by common method variance. Although the present study provides a significant and innovative contribution to the relationship between bullying and its different negative health consequences, future research should also explore other variables related to the workplace bullying, as well as dispositional variables that could potentially moderate bullying. For instance, it could be examined how workplace bullying could impact on the family domain and if the latter (e.g., family support; Lo Presti, D’Aloisio, & Pluviano, 2016) could act as a buffer with respect to negative consequences. Although supported by available evidence (Mikkelsen & Einarsen, 2002; Nielsen & Einarsen, 2012), the potential overlap between anxiety/depression and psychological negative symptoms could not be properly examined, for instance by means of confirmatory factor analysis, cause of limited sample size. Finally, data were collected when victims contacted the clinic center for their first consultations. Future research could examine if receiving subsequent therapy for the treatment of anxiety and/or depression could alter their subsequent association with physical/psychological negative symptoms.
